# Density Functional Theory Study of CO_2_ Hydrogenation on Transition-Metal-Doped Cu(211) Surfaces

**DOI:** 10.3390/molecules28062852

**Published:** 2023-03-22

**Authors:** Yushan Wang, Mengting Yu, Xinyi Zhang, Yujie Gao, Jia Liu, Ximing Zhang, Chunxiao Gong, Xiaoyong Cao, Zhaoyang Ju, Yongwu Peng

**Affiliations:** 1 College of Chemical & Material Engineering, Quzhou University, Quzhou 324000, China; lucky11022022@163.com (Y.W.); xinyiz@qzc.edu.cn (X.Z.); gaoyj0111@163.com (Y.G.); angela13lj@163.com (J.L.); jzy@qzc.edu.cn (Z.J.); 2 College of Biosystems Engineering and Food Science, Zhejiang University, Hangzhou 310058, China; zhangximing@zju.edu.cn (X.Z.); gongchunxiao@zju.edu.cn (C.G.); 3 Institute of Zhejiang University—Quzhou, Quzhou 324000, China; caoxy@zju.edu.cn; 4 College of Materials Science and Engineering, Zhejiang University of Technology, Hangzhou 310014, China

**Keywords:** CO_2_ hydrogenation, Cu-based catalyst, Bader charge, DFT

## Abstract

The massive emission of CO_2_ has caused a series of environmental problems, including global warming, which exacerbates natural disasters and human health. Cu-based catalysts have shown great activity in the reduction of CO_2_, but the mechanism of CO_2_ activation remains ambiguous. In this work, we performed density functional theory (DFT) calculations to investigate the hydrogenation of CO_2_ on Cu(211)-Rh, Cu(211)-Ni, Cu(211)-Co, and Cu(211)-Ru surfaces. The doping of Rh, Ni, Co, and Ru was found to enhance CO_2_ hydrogenation to produce COOH. For CO_2_ hydrogenation to produce HCOO, Ru plays a positive role in promoting CO dissociation, while Rh, Ni, and Co increase the barriers. These results indicate that Ru is the most effective additive for CO_2_ reduction in Cu-based catalysts. In addition, the doping of Rh, Ni, Co, and Ru alters the electronic properties of Cu, and the activity of Cu-based catalysts was subsequently affected according to differential charge analysis. The analysis of Bader charge shows good predictions for CO_2_ reduction over Cu-based catalysts. This study provides some fundamental aids for the rational design of efficient and stable CO_2_-reducing agents to mitigate CO_2_ emission.

## 1. Introduction

Since the industrial revolution, the massive emission of carbon dioxide (CO_2_) has caused a series of environmental problems and social issues. Therefore, the reduction and utilization of CO_2_ have drawn great attention from scientists [1,2,3]. There are three methods for the catalytic transformation of CO_2_ into value-added chemicals: thermal catalysis, electrocatalysis, and photocatalysis [4,5,6]. As one kind of inert molecule, CO_2_ is thermodynamically and kinetically stable due to its high C=O bond energy (750 kJ/mol). Generally, high temperatures are required for the utilization of CO_2_ [7]. The hydrogenation of CO_2_ into value-added chemicals can provide a sustainable pathway for its utilization [8,9]. The problems of hydrogen storage and transportation have been not only solved, but also, the valuable carbon-based resources have been effectively utilized [10]. For the various hydrogenation products, methanol is one of the most precious chemicals and has been widely used in automobiles, national defense, biomedicine, and so on [11,12].

Among the effective catalysts for CO_2_ reduction, Cu-based catalysts had been considered one of the most suitable catalysts due to their excellent catalytic activity and stability for CO_2_ hydrogenation for methanol production [13,14]. Liu et al. demonstrated through DFT and experiments that Cu^0^ species are active sites for CO_2_ hydrogenation to methanol when Cu_4_ supported on Al_2_O_3_ was used as the catalyst [15]. The study by Wu et al. [16] showed that Cu(211) with pre-adsorbed formate successfully confirmed its status as a major intermediate in the subsequent production of methanol. The hexagonal Cu(111) monolayer was considered as an efficient and selective catalyst for CO_2_ hydrogenation to CH_3_OH because of its strong nucleophilic nature compared to bulk Cu-based and Cu nanocluster-based catalysts [17].

Generally, the activity, selectivity, and stability of the active components have certain limitations. Therefore, it is crucial to select promoters to improve the selectivity, catalytic activity, and stability of the target products. For example, the adsorption, activation, and reduction of CO_2_ over Fe_x_/Cu(100) (x = 1–9) were investigated, and the calculations showed that the doped Fe on the pure Cu(100) surface can improve the adsorption of CO_2_ and enhance CO_2_ activation [18]. Liu et al. [19] reported that the addition of Pd, Rh, Pt, and Ni metals into Cu catalysts can facilitate the production of methanol. Additionally, the optimal CuNi alloy supported on the CeO_2_ nanotube catalyst showed a CO_2_ conversion of 17.8% [14].

As one of the crucial elementary reaction steps for the utilization of CO_2_ into value-added chemicals, the activation of CO_2_ plays a critical role in the whole process. Currently, three pathways have been proposed, which are the direct dissociation of CO_2_, formate (HCOO) pathways, and carboxylate (COOH) pathways [20,21]. Tang et al. [22] proposed that the Ga–Ni(211) surface prefers CO_2_ hydrogenation, whereas Ni(211) is more favorable for the dissociation of CO_2_. For the Cu-ZnO-Al_2_O_3_ catalysts, HCOO is an intermediate species for the synthesis of methanol [21]. However, Graciani et al. [23] proposed that COOH is an intermediate for the synthesis of methanol on the highly active CeO_X_-Cu(111) catalysis. Theoretical calculations showed that HCOO is an intermediate species for the synthesis of methanol on the Cu(111) surface, and the hydrogenation reaction of HCOO and H_2_COO is a rate-determining step [24]. Additionally, the optimal path for CO_2_ hydrogenation to CH_3_OH is CO_2_*→HCOO*→HCOOH*→H_2_COOH*→CH_3_O*→CH_3_OH* on the PdCu(111) surface [25]. Zhang et al. [26] believed that methanol is the dominant product via mono-HCOO intermediate on the Cu(111), Cu(100), Cu(111), Cu(111), Cu(111), and Cu(211) surfaces. Moreover, they proposed that the catalytic performance of CO_2_ activation and conversion could be effectively tuned by adjusting defect site types.

The catalytic performance is attributed to the structure of the catalyst’s surface [27,28]. Therefore, an in-depth understanding of the surface structure of the catalyst is of great significance to improving the performance of catalysts. As an effective computational chemistry method, density functional theory (DFT) has been widely used in the study of microscopic reaction mechanisms on the surface of catalysts [29,30]. It has been reported that metal surfaces are not always perfect under realistic conditions [31]. Previous studies have shown that stepped surfaces exhibit better catalytic activity than flat surfaces [32]. Compared to the flat Rh(111) surface, the stepped Rh(211) surface exhibits a lower activation barrier for CO dissociation [33]. In addition, the stepped Cu(211) surface is more favorable for the hydrogenation of CO_2_ than the flat Cu(111) surface [34]. Therefore, the stepped Cu(211) surface was chosen to study CO_2_ hydrogenation over Cu catalysts.

In this work, we investigated the effect of transition metal dopants on a Cu(211) surface for CO_2_ activation by using DFT calculations. The research started with the investigation of the stability of Cu(211)-M(Rh, Ni, Co, Ru) surfaces followed by the adsorption structure and energy of intermediates on the pure Cu(211) and Cu(211)-M(Rh, Ni, Co, Ru) surfaces. Then, the activation barriers and reaction energies of the H_2_ dissociation and CO_2_ activation were calculated. Furthermore, differential charge density and Bader charge analysis were analyzed to elucidate the charge transfer and interaction between M (Rh, Ni, Co, Ru) and Cu surfaces. This will provide some help in understanding the mechanism of conversion of CO_2_ and in designing more effective catalysts in the theoretical views.

## 2. Results and Discussion

### 2.1. Formation Energies of Cu(211)-M Surfaces

To evaluate the stability of the forming surface, the formation energy was introduced [22]. Table 1 reports the formation energies of Cu(211)-M (M = Rh, Ni, Co, Ru) surfaces. The calculated formation energies for Cu(211)-Rh, Cu(211)-Ni, Cu(211)-Co, and Cu(211)-Ru surfaces are −2.75, −1.62, −2.28, and −4.02 eV, respectively. This clearly shows that it is favorable to exchange an M (M = Rh, Ni, Co, Ru) surface atom for a Cu atom in the Cu(211) model. The substitution of a Ru atom is most favorable because of the most negative formation energy.

### 2.2. Adsorption of Intermediates on Cu(211)-M Surfaces

**Figure 1:**
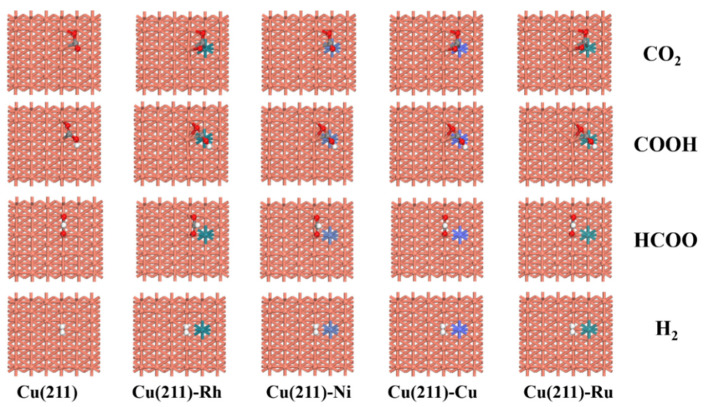
The stable adsorption configurations of CO_2_, COOH, HCOO, and H_2_ on the pure and transition-metal-doped Cu(211) (M = Rh, Ni, Co, and Ru) surfaces.

To gain fundamental insights into M (M = Rh, Ni, Co, Ru) on reactivity, the adsorption of all possible species involved in CO_2_ hydrogenation was examined [35,36]. Firstly we calculated the adsorption energy and corresponding adsorption configurations of CO_2_, H_2,_ COOH, and HCOO on the Cu(211)-M (M = Rh, Ni, Co, Ru) surface. Table 2 lists the adsorption energies of the most stable adsorbed states on these surfaces. The corresponding adsorption configurations are presented in Figure 1. For CO_2_ adsorption, the order of adsorption energy is Cu(211) < Cu(211)–Rh < Cu(211)–Ni < Cu(211)–Ru < Cu(211)–Co. For COOH adsorption, compared to that of on the pure Cu(211) surface (−1.76 eV), the adsorption energy is lowered by 0.61, 0.29, 0.59, and 0.85 eV on the Rh-, Ni-, Co-, and Ru-doped surfaces, respectively. Therefore, the addition of transition metals facilitates the formation of COOH intermediates. For HCOO adsorption, the order of adsorption energy is Cu(211)–Rh < Cu(211)–Ru < Cu(211)–Ni < Cu(211)–Co = Cu(211). For H_2_ adsorption on the pure Cu(211) surface, H_2_ has the strongest adsorption, with an energy of −0.30 eV, while on the Cu(211)-Co and Cu(211)-Ru surfaces, H_2_ has the weakest adsorption, with an energy of −0.01 eV. Thus, H_2_ adsorption is inhibited by Rh, Ni, Co, and Ru doping. To further understand the interaction between CO_2_, COOH, HCOO, and H_2_ species and the surface, Bader charge analysis was introduced. It was reported that the higher the net Bader charge, the more negative adsorption energy of CO_2_ [37]. A similar conclusion can be drawn in our work. As shown in Table 2, on the Cu(211) surface, the weakest adsorption of CO_2_ was observed due to the lowest net Bader charge. Conversely, the strongest energy of HCOO was attributed to the highest net Bader charge.

### 2.3. H_2_ Dissociation

**Figure 2:**
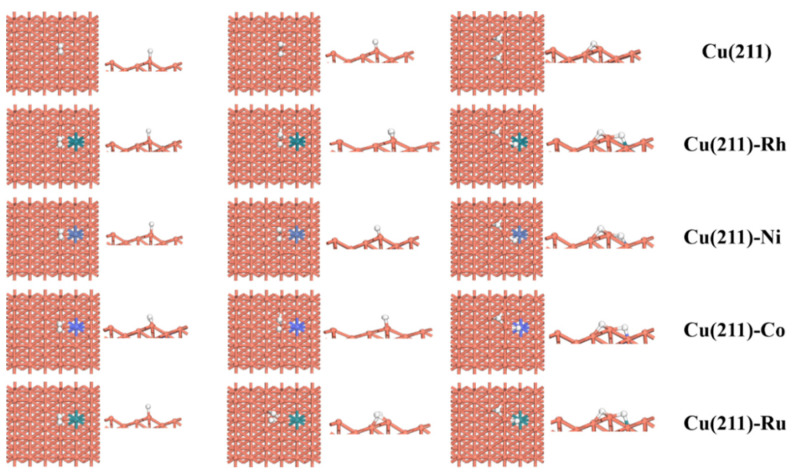
The stable adsorption configurations of CO_2_, COOH, HCOO, and H_2_ on pure and transition-metal-doped Cu(211)-M (M = Rh, Ni, Co, and Ru) surfaces.

For the hydrogenation and activation of CO_2_, the dissociation of H_2_ is the key initial step [26]. To investigate the H_2_ dissociation on the catalyst surface, the activation barrier and reaction energy of H_2_ dissociation on the Cu(211)-Rh, Cu(211)-Ni, Cu(211)-Co, and Cu(211)-Ru surfaces were calculated and are shown in Table 3. The corresponding geometries of the initial state (IS) of H_2_ adsorption, transition state (TS), and final state (FS) for H_2_ dissociation on the pure and transition-metal-doped Cu(211) surfaces are summarized in Figure 2.

As shown in Figure 2, it can be found that H_2_ prefers to adsorb at the top site on these five surfaces. After the dissociation of H_2_ on the Cu(211) surface, two H atoms can be adsorbed on the adjacent 3F site; for Cu(211)-Rh, Cu(211)-Ni, Cu(211)-Co, and Cu(211)-Ru surfaces, two H atoms are adsorbed on 3F and bridge. For the dissociation of H_2_, the activation energy barrier on Cu(211) is 0.44 eV, which is consistent with the previously reported literature and differs slightly from 0.09 eV [26]. Apparently, Co and Ru doping promote the H–H bond scission, and the barrier is lower by 0.25 and 0.22 eV. On the Cu(211)-Rh and Cu(211)-Ni surfaces, the dissociation of H_2_ required to overcome the energy barrier is approximately 0.42 eV. Therefore, there is a slight effect on Cu(211) surface. In addition, the reaction energies of H_2_ dissociation on the Cu(211)-Rh, Cu(211)-Ni, Cu(211)-Co, and Cu(211)-Ru surfaces are −0.84, −0.57, −0.86, and −1.01 eV, respectively. Therefore, the values of *E*_r_ on all the surfaces suggest that the elementary step is exothermic. Tang et al. [22] reported that the existing adsorption form of H_2_ is dissociative adsorption on the Ni(211) and Ga–Ni(211) surfaces. More importantly, it can also be found that H_2_ is easily activated and dissociated into adsorbed H (H*). The H* is the main form of H_2_ on these five surfaces.

### 2.4. CO_2_ Activation

The activation of CO_2_ is a key step in many catalytic reactions [38,39]. Therefore, it is very important to study the mechanism of CO_2_ activation. For CO_2_ activation, H-assisted dissociation via carboxyl (COOH) and formate (HCOO) intermediates have been taken into consideration on the Cu(211)-Rh, Cu(211)-Ni, Cu(211)-Co, and Cu(211)-Ru surfaces. The activation barriers and the reaction energies for CO_2_ activation on the pure and M-doped Cu(211)(M = Rh, Ni, Co, and Ru) surfaces are summarized in Table 4 and Table 5.

**Figure 3:**
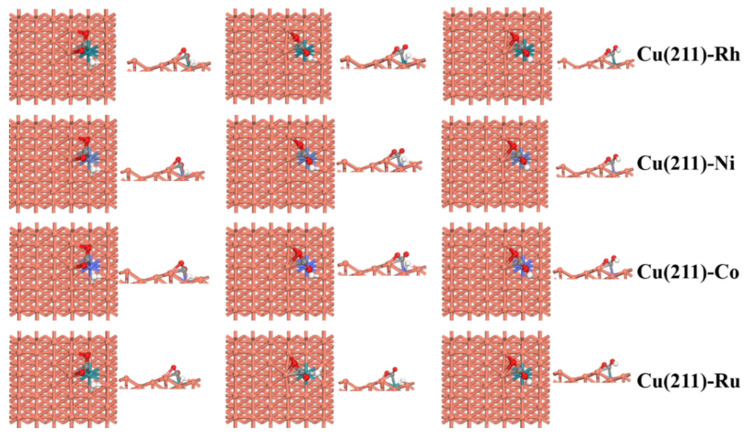
Top and side views of the initial state (IS), transition state (TS), and final state (FS) for CO_2_ activation via COOH intermediate on the pure and transition-metal-doped Cu(211)-M (M = Rh, Ni, Co, and Ru) surfaces.

As shown in Figure 3, in the initial state, the V-type adsorbed CO_2_ molecules and H atoms were coadsorbed on the surface of the catalyst. CO_2_ was adsorbed on the 4F active site, and H preferred to adsorb at the 3F site. When the reaction occurred, H moved to the O atom to form COOH, and COOH adsorbed on the 4F site. From Table 4, the activation barrier of CO_2_ hydrogenation is in the order Cu(211)-Ru < Cu(211)-Ni < Cu(211)-Rh < Cu(211)-Co < Cu(211). Thus, Co, Rh, Ni, and Ru doping promotes O-H bond formation and lowers the barrier by 0.81, 0.86, 0.88, and 0.95 eV, respectively. In addition, the reaction energy of CO_2_ activation all are exothermic by 0.40 eV on the M-doped Cu(211)(M = Rh, Ni, Co, and Ru) surfaces. The above analysis, it clearly shows that Co, Rh, Ni, and Ru doping promotes CO_2_ activation to form COOH.

**Figure 4:**
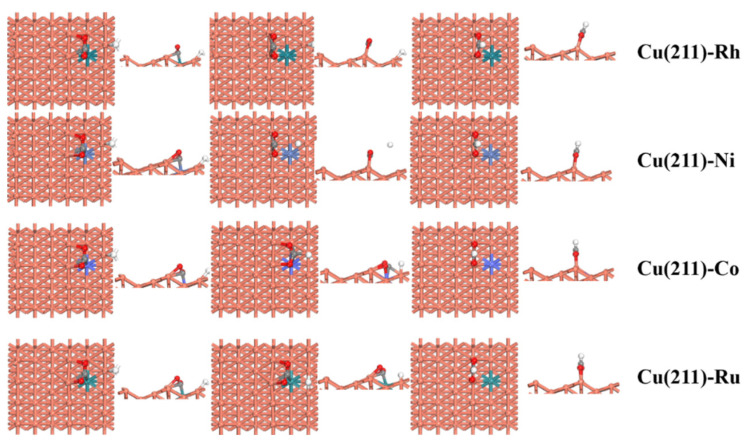
Top and side views of the initial state (IS), transition state (TS), and final state (FS) for CO_2_ activation via HCOO intermediate on the pure and transition-metal-doped Cu(211)-M (M = Rh, Ni, Co, and Ru) surfaces.

**Figure 5:**
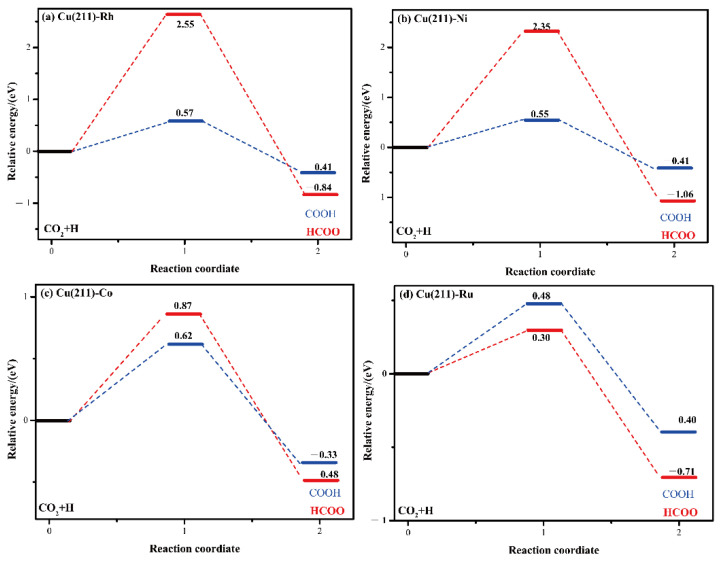
Energy profiles for CO_2_ hydrogenation to COOH and HCOO on (**a**) Cu(211)-Rh, (**b**) Cu(211)-Ni, (**c**) Cu(211)-Co, and (**d**) Cu(211)-Ru surfaces.

In the initial state, CO_2_ is adsorbed on the 4F active site and H prefers to adsorb at 3F site. When the reaction occurred, H moved to the C atom to form HCOO and HCOO adsorbed on the bridge site, as shown in Figure 4. From Table 5, compared to the pure Cu(211) [26] surface (0.74 eV), the activity of CO_2_ activation to HCOO is higher on the Cu (211)-Ru (0.30 eV). Conversely, the C–H bond formation is inhibited by the Rh/Ni/Co doping, with the barrier being raised to 2.55, 2.35, and 0.87 eV, respectively. In addition, the reaction energy of CO_2_ activation is exothermic on the M-doped Cu(211)(M = Rh, Ni, Co, and Ru) surfaces. In summary, this is different from the formation of COOH—only Ru additive promotes the production of HCOO. Figure 5 it clearly shows that CO_2_ hydrogenation to COOH is more plausible on the Cu(211)-Rh, Cu(211)-Ni, and Cu(211)-Co surfaces, while CO_2_ hydrogenation to HCOO is more preferable on the Cu(211)-Ru surface.

### 2.5. Electronic Structure Analysis

**Figure 6:**
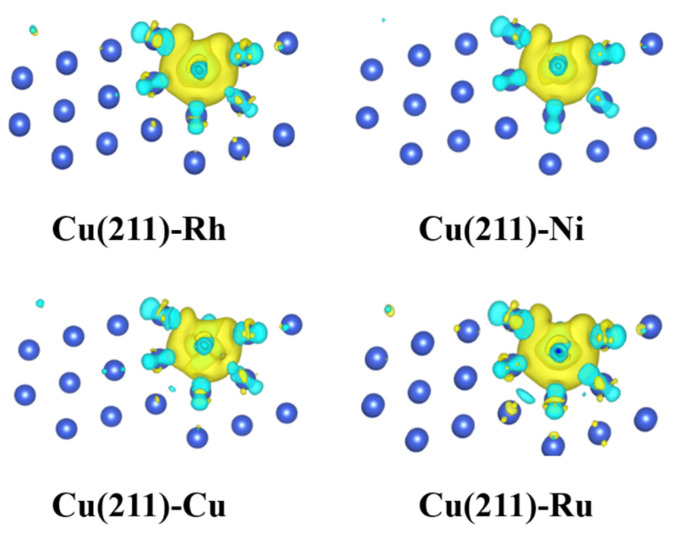
Side views of differential charge density distribution for M atoms (M = Rh, Ni, Co, and Ru) on Cu(211)-M surfaces. The yellow and blue regions represent charge accumulation and depletion, respectively.

Generally, the catalytic performance is attributed to the electronic properties [28,40,41]. The addition of a small number of additives could change the morphology of the catalyst or modify the electronic properties of the active phase metal Cu. To visualize the electronic interaction between M (M = Rh, Ni, Co, and Ru) and Cu surfaces, the differential charge density distribution of the M/Co systems is shown in Figure 6. The results showed that the doping of Rh, Ni, Co, and Ru modified the electronic properties of Cu and therefore affected the activity of Cu-based catalysts. The charge transfer between Co surfaces and M-doped surfaces was quantified using Bader charge analysis, which is listed in Table 1. The results show that the localized electron is transferred from the Cu surface to Rh, Ni, and Ru atoms, which is attributed to the fact that Rh, Ni, and Ru are more electronegative than Cu. In contrast, the localized electron is transferred from the Co atom to the Cu surface because of the lower electronegativity of Co.

**Figure 7:**
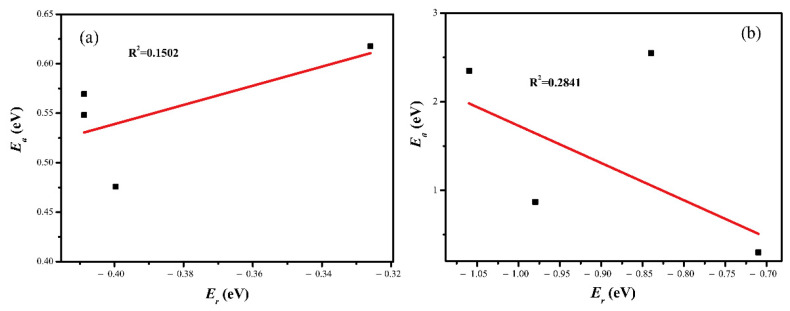
Relationship between the activation barrier and the reaction energy via (**a**) CO_2_+H→COOH and (**b**) CO_2_+H→HCOO.

Exploring CO_2_ reduction on catalysts is a very complicated and comprehensive work, and we attempt to find descriptors that can predict activation energy in this part. Based on the computed energy data on the pure and M-doped Cu(211) surfaces (M = Rh, Ni, Co, and Ru), we examined the Brønsted–Evans–Polanyi (BEP) [42], which is the most successful example of the relationship between the associated activation barrier and reaction energy. In the previous studies, Chen et al. [41] found that the reaction energy can be a descriptor for the CO activation on different χ-Fe_5_C_2_ catalyst surfaces. Gong et al. [37] reported that there is a linear relationship between the CO activation barrier and reaction energy on the pure and M-doped Fe(100) surfaces (M = Cr/Mn/Co/Ni/Cu). Firstly, we analyze the relationship between the activation barrier and the reaction energy of CO_2_ hydrogenation to COOH and HCOO on these five doped surfaces. The correlation is shown in Figure 7; it can be found that the reaction energy of CO_2_ reduction does not give a good description of the CO_2_ activation barrier for the different transition metal dopants’ Cu(211) surfaces. In addition, Chen et al. [41] suggested that the corresponding linear relation is slightly poor between the activation barrier and the reaction energy for CO dissociation on the different χ-Fe_5_C_2_ surfaces.

**Figure 8:**
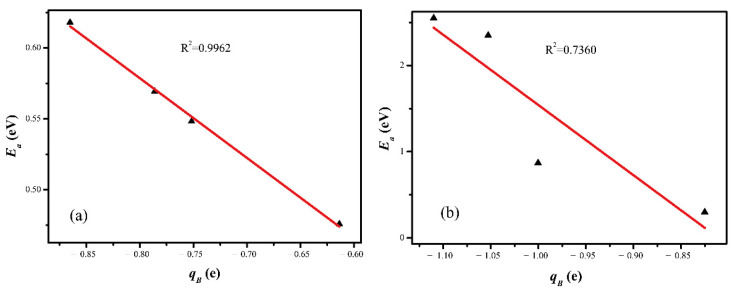
Trends in the CO_2_ activation barrier (*E*_a_) as a function of the average Bader charge (q_B_) of the involved surface Cu and doped metal atoms for the CO_2_ hydrogenation to (**a**) COOH and (**b**) HCOO.

In order to gain insight into the underlying mechanism of electronic effects introduced by transition metals for CO_2_ reduction, the Bader analysis is employed. Figure 8 shows that the charges of the involved surface Cu and doped metal atoms follow a nearly linear relation with the CO_2_ activation barrier. Obviously, different transition metal dopants’ Cu surfaces have different abilities to donate electrons for the CO_2_ activation. Therefore, the atomic charge of the involved surface and doped metal atoms for the CO_2_ activation is suggested as a dominant factor to describe the CO_2_ activation on the different Cu-based catalyst surfaces. Therefore, we could predict the reactivity of CO_2_ reduction on the Cu surfaces with these correlations.

As mentioned above, Ru has been shown to be the most effective additive for CO_2_ hydrogenation in Cu-based catalysts. It can be argued that Ru additives can improve the catalytic activity of copper-based catalysts in several ways. First, electron transfer has been reported to be essential for reactant adsorption, which in turn affects the activity of reactants [43]. Ru alters the electronic properties of Cu, thus influencing the charge of surface reactants. Moreover, Ru is an effective catalyst for CO_2_ hydrogenation [44]. Wesselbaum et al. suggested that a single Ru-triphos catalyst could improve the hydrogenation of CO_2_ to methanol via the direct route [45]. Thus, the addition of Ru facilitates the conversion of CO_2_.

## 3. Materials and Methods

### 3.1. Model

**Figure 9:**
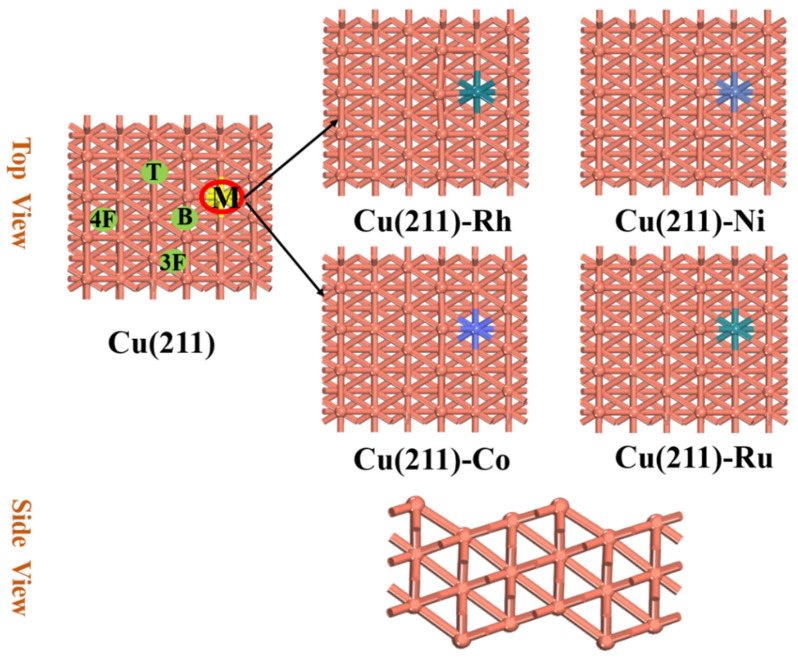
Top and side views of structures of pure Cu(211) surface and transition-metal-doped Cu(211)-M (M = Rh, Ni, Co, and Ru) surfaces and possible adsorption sites: top site (T), bridge site (B), three-fold site (3F), and four-fold site (4F).

In order to study the CO_2_ hydrogenation reaction mechanism using doped metals on the Cu-based catalysts, we selected a p(2×4) Cu(211) periodic model with three layers, which included 72 Cu atoms. There were different adsorption sites on the surface of Cu(211), including top (T), bridge (B), three-fold (3F), and four-fold (4F) sites, which are shown in Figure 9. Additionally, there was no interaction between the periodically repeated models. The vacuum layer was set to 15 Å. During the calculations, the adsorbates and top two layers were relaxed, and the remaining bottom layers were fixed in their bulk positions. As shown in Figure 9, the substitution model was used, in which the local surface Cu sites are replaced by Rh, Ni, Co, and Ru. The formula for formation energy is as follows [22]:

*E_f_* = *E*_Cu(211)-M_* + E_Cu_ − E*_M_
*−*
*E*_Cu(211)_ (1)

where *E*_sub_ is the substitution energy of the Cu(211)-M surface; *E*_Cu(211)_ and *E*_Cu(211)-M_ are the total energies of Cu(211) and Cu(211)-M surfaces, respectively. *E*_Cu_ and *E*_M_ are the total energies of single Cu and promoter atoms (including Rh, Ni, Co, and Ru). According to this definition, it is indicated that the negative *E*_f_ value suggests that the formation process is exothermic and preferable.

### 3.2. Calculation Method

The VASP (Vienna Ab-initio Simulation Package) software (version 5.4.4) developed by the University of Vienna Hafner was used to study the adsorption energies and activation energies of the CO_2_ hydrogenation [46,47]. The generalized gradient approximation (GGA) method with Perdew–Burke–Ernzerhof (PBE) was used as the exchange–correlation energy. [48] The plane wave basis set was set to 400 eV. When the total energy converges to 10^−5^ eV and the force is less than 0.03 eV/Å, the geometry optimization is thought to be converged. A 3 × 2 × 1 k-point sampling in the surface Brillouin zone was used for all calculations. We tested the parameters including k-point grids and cutoff energy (see Table 6) for convergence accuracy using COOH adsorption on a Cu(211)-Ru surface as an example, and the results showed energy differences in the range of 0.01–0.04 eV. The CI-NEB (climbing image-nudged elastic band) [49,50] was performed to confirm the transition state structure. When atomic force is less than 0.05 eV/Å, the transition state would be converged. In addition, the vibrational frequencies were introduced to verify the transition states with only one imaginary frequency. The adsorption energy (*E*_ads_) of adsorbates is defined as:

*E*_ads_ = *E*_adsorbate/slab_ − *E*_slab_ − *E*_adsorbate_ (2)

here, *E*_adsorbate/slab_, *E*_slab_, and *E*_adsorbate_ are the total energies of the slab with the adsorbate, the slab surface, and the free adsorbate, respectively. It is indicated that the more negative the value of *E*_sub_, the stronger the adsorption. The activation barrier (*E*_a_) and reaction energy (*E_r_*) are defined as:

*E_a_ = E_TS_ − E_IS_* (3)

*E_r_ = E_FS_ − E_IS_* (4)

here, *E_IS_, E_TS_*, and *E_FS_* are the total energy of the initial, transition, and final states.

## 4. Conclusions

In this work, the effects of transition metal doping on Cu(211) surfaces for CO_2_ hydrogenation were investigated using the density functional theory method. It is revealed that the doping of Rh, Ni, Co, and Ru doping enhances the dissociation of H_2_ and the hydrogenation of CO_2_ to COOH. For the hydrogenation of CO_2_ to HCOO, Ru shows a positive role in promoting the formation of HCOO, while the doping of Rh, Ni, and Co leads to an increase in the energy barrier. Therefore, the doping of Ru is the most effective for the reduction of CO_2_. Differential charge analysis showed that the doping of Rh, Ni, Co, and Ru alters the electronic properties of Cu, which in turn influences the activity of Cu-based catalysts for CO_2_ reduction. Bader charge as a descriptor was introduced in CO_2_ activation on various Cu(211) surfaces. According to the calculations, there is a good relationship between the atomic charges of the involved surface Cu and M (M = Rh, Ni, Co, and Ru) atoms and the activation barriers for CO_2_ activation. With these correlations, the performance of different Cu-based catalysts could be reasonably and accurately predicted.

## Figures and Tables

**Table 1:** Formation energies (eV) and Bader charges (q, e) of transition-metal-doped Cu(211)-M (M = Rh, Ni, Co, Ru) surfaces.

Surface	Formation Energy	q
Cu (211)-Rh	−2.75	−0.32
Cu (211)-Ni	−1.62	−0.03
Cu (211)-Co	−2.28	0.02
Cu (211)-Ru	−4.02	−0.16

**Table 2:** Adsorption energies (*E*_ads_, eV) and net Bader charges (q, e) of CO_2_, COOH, HCOO, and H_2_ on pure Cu(211) and transition-metal-doped Cu(211)-M (Rh, Ni, Co, Ru) surfaces.

	CO_2_		COOH		HCOO		H_2_	
Surface	*E*_ads_	q	*E*_ads_	q	*E*_ads_	q	*E*_ads_	q
Cu(211)	−0.26	0.80	−1.76	0.39	−3.32	0.65	−0.30	0.02
Cu(211)-Rh	−0.31	0.84	−2.37	0.51	−3.16	0.61	−0.18	0.01
Cu(211)-Ni	−0.34	0.85	−2.05	0.43	−3.20	0.62	−0.11	−0.02
Cu(211)-Co	−0.38	0.97	−2.35	0.50	−3.32	0.65	−0.01	−0.02
Cu(211)-Ru	−0.35	0.91	−2.61	0.55	−3.18	0.61	−0.01	−0.02

**Table 3:** The activation barrier (eV) and reaction energy (eV) of H_2_ dissociation on pure Cu(211) and transition-metal-doped Cu(211)-M (M = Rh, Ni, Co, Ru) surfaces together with the H–H bond length (*d*_H-H_/Å) in the transition state and the corresponding imaginary frequency of the transition state *v*(cm^−1^).

Surface	Activation Barrier	Reaction Energy	*d*_H-H_	*v*(cm^−1^)
Cu(211) [26]	0.44	−0.51	1.3349	1054*i*
Cu (211)-Rh	0.41	−0.84	1.321	1186*i*
Cu (211)-Ni	0.42	−0.57	1.307	1254*i*
Cu (211)-Co	0.19	−0.86	1.320	1130*i*
Cu (211)-Ru	0.22	−1.01	0.969	465*i*

**Table 4:** The activation barrier (eV) and reaction energy (eV) of carbon dioxide hydrogenation via COOH intermediate on the pure and transition-metal-doped Cu(211)-M (M = Rh, Ni, Co, and Ru) surfaces together with the C–H bond length (*d*_C-H_/Å) in the transition state and the corresponding imaginary frequency of the transition state *v*(cm^−1^).

Surface	Activation Barrier	Reaction Energy	*d_C_*_-H_	*v*(cm^−1^)
Cu(211)	2.02	0.50	/	/
Cu (211)-Rh	0.57	−0.41	1.481	1189*i*
Cu (211)-Ni	0.55	−0.41	1.491	1270*i*
Cu (211)-Co	0.62	−0.33	1.467	1254*i*
Cu (211)-Ru	0.48	−0.40	2.362	1070*i*

**Table 5:** The activation barrier(eV) and reaction energy (eV) of carbon dioxide hydrogenation via HCOO intermediate on the pure and transition-metal-doped Cu(211)-M (M = Rh, Ni, Co, and Ru) surfaces together with the C–H bond length (*d*_C-H_/Å) in the transition state and the corresponding imaginary frequency of the transition state *v*(cm^−1^).

Surface	Activation Barrier	Reaction Energy	*d_C_*_-H_	*v*(cm^−1^)
Cu(211) [26]	0.74	0.46	/	/
Cu(211)-Rh	2.55	−0.84	4.994	1001.5*i*
Cu(211)-Ni	2.35	−1.06	3.206	498.5*i*
Cu(211)-Co	0.87	−0.98	1.820	867.7*i*
Cu(211)-Ru	0.30	−0.71	2.721	1160*i*

**Table 6:** Model testing parameters for COOH adsorption on the Cu(211)-Ru surface.

Surface Slabs	Cut-Energy	k-Points	*E*_ads_ (eV)
Cu(211)-Ru	400	3 × 2 × 1	−2.61
	400	3 × 3 × 1	−2.57
	400	4 × 4 × 1	−2.59
Cu(211)-Ru	400	3 × 2 × 1	−2.61
	500	3 × 2 × 1	−2.57
	600	3 × 2 × 1	−2.57

## Data Availability

Data can be found in the manuscript.

## Sample Availability

Samples of the compounds are not available from the authors.
